# Attenuated reovirus displays oncolysis with reduced host toxicity

**DOI:** 10.1038/sj.bjc.6606053

**Published:** 2010-12-21

**Authors:** M Kim, K A Garant, N I zur Nieden, T Alain, S D Loken, S J Urbanski, P A Forsyth, D E Rancourt, P W K Lee, R N Johnston

**Affiliations:** 1 Department of Medical Sciences, University of Calgary, Calgary, Alberta, Canada; 2 Department of Microbiology & Immunology, Dalhousie University, Halifax, Nova Scotia, Canada; 3 Department of Biochemistry & Molecular Biology, University of Calgary, Faculty of Medicine, Calgary, Alberta, Canada; 4 Department of Pathology, University of Calgary, Calgary, Alberta, Canada

**Keywords:** mammalian reovirus, attenuated, persistent infection, oncolysis, reduced toxicity, sigma1

## Abstract

**Background:**

Although the naturally occurring reovirus causes only mild symptoms in humans, it shows considerable potential as an oncolytic agent because of its innate ability to target cancer cells. In immunocompromised hosts, however, wild-type reovirus can target healthy tissues, including heart, liver, pancreas and neural structures.

**Methods:**

We characterized an attenuated form of reovirus (AV) derived from a persistently infected cell line through sequence analysis, as well as western blot and *in vitro* transcription and translation techniques. To examine its pathogenesis and oncolytic potential, AV reovirus was tested on healthy embryonic stem cells, various non-transformed and transformed cell lines, and in severe combined immunodeficiency (SCID) mice with tumour xenografts.

**Results:**

Sequence analysis of AV reovirus revealed a premature STOP codon in its sigma 1 attachment protein. Western blot and *in vitro* translation confirmed the presence of a truncated *σ*1. In comparison to wild-type reovirus, AV reovirus did not kill healthy stem cells or induce black tail formation in SCID mice. However, it did retain its ability to target cancer cells and reduce tumour size.

**Conclusion:**

Despite containing a truncated attachment protein, AV reovirus still preferentially targets cancer cells, and compared with wild-type reovirus it shows reduced toxicity when administered to immunodeficient hosts, suggesting the potential use of AV reovirus in combination cancer therapy.

Mammalian REO (respiratory enteric orphan) virus is a small, non-enveloped icosahedral virus that contains segments of double-stranded RNA as its genome. In humans, reovirus targets the upper respiratory and gastrointestinal tracts to generate a relatively benign and often asymptomatic infection (Tyler, 2001). Of interest is its ability to infect and kill many types of transformed cells (Hashiro *et al*, 1977; Duncan *et al*, 1978), making reovirus one example among a variety of replication competent oncolytic viruses that potentially serve as anti-cancer therapies. Such oncolytic viruses have, in principle, two main advantages over conventional chemotherapy and radiotherapy. First, they generally target cancer cells because of their reduced ability, whether innate or engineered, to replicate in normal cells. Second, in comparison with replication-incompetent vectors, they can propagate from initially infected cancer cells to surrounding or distant cancer cells, thereby achieving a large volume of distribution and potent anti-cancer effects. Despite the above, ongoing challenges in this field include, ensuring that (a) all cancer cells are efficiently targeted, including those that have metastasised to distant sites; (b) damage to normal tissues and proliferating cells is minimized and (c) natural host-immune responses do not prematurely block viral eradication of tumour cell populations.

Reovirus Type 3 Dearing (T3D) is a naturally occurring oncolytic virus that preferentially targets Ras-transformed cells both *in vitro* and *in vivo* (Coffey *et al*, 1998; Strong *et al*, 1998; Norman *et al*, 2004). Constitutively activated Ras contributes to reovirus oncolysis by increasing the efficiency of (1) reovirus disassembly during entry, (2) infectious virion production and (3) apoptosis and virion release (Marcato *et al*, 2007). As *Ras* gene mutations are observed in over 30% of all human cancers (Duursma and Agami, 2003), these findings have led to the current use of reovirus in phase I, II and III clinical trials (Oncolytics Biotech Inc., 2010). However, in immunocompromised hosts, such as newborn and severe combined immunodeficiency (SCID) animals; wild-type reovirus shows significant pathogenicity, especially to neural and cardiac muscle tissues (Sabin, 1959; Weiner *et al*, 1977; Baty and Sherry, 1993; Loken *et al*, 2004). Occasionally, this pathogenesis is observed in immunocompetent hosts, but mainly at high viral loads (Hirasawa *et al*, 2003; Terheggen *et al*, 2003). Clinical trials have reported no severe toxicity when utilising wild-type reovirus in combination with radiation or chemotherapy (Thirukkumaran and Morris, 2009). Even so, a less virulent reovirus that displays reduced pathogenicity to healthy tissues and cells while retaining potent oncolytic activity could still be beneficial by providing a potentially higher number of treatments or dosage.

One way of generating less virulent reovirus is through the establishment of persistently infected cells. Persistent reovirus infection is occasionally induced in various cell types *in vitro* (Dermody, 1998). Although the underlying basis of reovirus persistent infection is not well understood, it has been speculated that interactions between the virus and host cell can be fundamentally altered such that a modified cytopathic virus–host relationship is established (Wetzel *et al*, 1997). Importantly, such infections can give rise to distinct reovirus variants (Ahmed and Fields, 1982; Wilson *et al*, 1996; Wetzel *et al*, 1997); however, the potential utility of these variants for reovirus oncolysis has yet to be examined. In this report, we describe a mutant reovirus with a modified *S1* gene that has been isolated from persistently infected cells. The modified reovirus contains a truncated *σ*1-cell-attachment protein and displays significantly reduced pathogenic potential to healthy cells while maintaining its oncolytic activity. This attenuated virus (AV) may provide a desirable option in future oncolytic cancer therapy.

## Materials and methods

### Cell lines

HT1080 human fibrosarcoma, L929 murine fibrosarcoma, Raji human Burkitt's lymphoma, CA46 human Burkitt's lymphoma and HEK 293 cell lines were purchased from the American Type Culture Collection (ATCC, Manassas, VA, USA) and were maintained according to the ATCC protocols. To establish persistently infected cell lines, HT1080, Raji and CA46 cells were infected with wild-type (WT) reovirus at a multiplicity of infection (MOI) of 20. The surviving cells were maintained for 3–10 weeks until they reached confluency and subsequently reinfected 2–3 times for up to 3–7 weeks to ensure the selection of virus-resistant cells (Ahmed and Fields, 1982). From the surviving cells of HT1080, several subclones were obtained by serial dilution. Clone HTR1 (HT1080 virally resistant clone 1) was used for this study. HTR1 cells were persistently infected and maintained for more than 48 months (Kim *et al*, 2007). From surviving Raji and CA46 cells, persistently infected Raji PI (Alain *et al*, 2006), and CA46 PI were stably established and maintained for more than 6 months.

Murine R1 and D3 embryonic stem cell lines were also purchased from the ATCC. The cells were maintained in high glucose DMEM, 15% FCS (selected batches), 50 U ml^−1^ penicillin and 50 *μ*g ml^−1^ streptomycin, 1% nonessential amino acids and 0.1 mM *β*-mercaptoethanol as described (zur Nieden *et al*, 2001). Pluripotency was sustained by adding 1000 U ml^−1^ leukaemia inhibitory factor (LIF) to the culture. The cultures were passaged at 80% confluency approximately every second day. All medium components were purchased from Invitrogen (Burlington, ON, Canada). Murine MES1 embryonic stem cells were isolated from 3.5-day postcoitum embryos of the 129 IM/SvJ substrain at the blastocyst stage. MES1 were routinely grown as described for R1 and D3 ESCs.

### Reovirus propagation, titration and infection

The wild-type reovirus T3D strain used in this study was propagated in L929 cells and purified by cesium chloride ultracentrifugation as described previously (Smith *et al*, 1969). Attenuated reovirus derived from the HTR1 culture was purified by the same method used in wild-type reovirus preparation, except AV reovirus was propagated in HT1080 and L929 cells. For AV reovirus collection from HT1080- and HTR1-infected supernatants (used only in Figure 2B), high-speed ultracentrifugation at 35 000 r.p.m. was used to pellet the virus.

For wild-type reovirus titration, HEK 293 cells were plated in 6-well plates at 2 × 10^5^ cells per well. After 2 h of adsorption at 37°C, the inoculum was removed. Cell monolayers were then covered with 1% agar and fresh medium. Plaques were counted 5–7 days after infection. For AV reovirus titration, the same procedure was followed except after 5–7 days of infection, the agar was removed and cell monolayers were fixed/permeabilzed with cytofix/cytoperm (BD Biosciences, Mississauga, ON, Canada) for immunostaining with reovirus antiserum and secondary FITC antibody. Attenuated reovirus plaques were identified by immunofluorescence detection. Furthermore, WT and AV reovirus titrations were also performed on monolayers of L929 cells, which after an initial infection for 1 h at 37°C, were subsequently covered with a 1 : 1 ratio of 1% agar and 2X MEM containing 10% horse serum. Plaques were counted 5–7 days after infection using a neutral red counterstain. In either case, titers yielded PFU:particle ratios of approximately 1 : 200 for both WT and AV reovirus, respectively.

Cells were infected with reovirus (MOI of 20 or 40) in serum-free media and after 1 h the virus-containing media was replaced with the appropriate cell-culture media.

### Sequencing of reovirus *S1* and *S4* genes

Viral dsRNAs, derived from WT reovirus and Raji, CA46 and HTR1 persistent reovirus infected cells were TRIzol extracted, converted to cDNA by reverse transcription, and PCR amplified. The PCR reactions were hot-started (held at 94°C for 30 s), followed by 35 cycles of 1 min at 94°C, 1 min at 59°C and 1 min 30 s at 72°C. A final extension for 10 min at 72°C was performed and the reaction product was cooled to 4°C. The primers used to amplify the *S1* gene segment were 5′-CATGAATTCATGGATCC TCGCCTACGTTAAGAAG-3′ (Forward) and 5′-CAGAAGCTTCTGATCCTCACGTGAA ACTACGC-3′ (Reverse), and the primers used to amplify the *S4* gene segment were 5′-CAAGAATTCTTGTCGCAATGGAGGTGTG-3′ (Forward) and 5′-TCTAAGCTTAGATG GGGGTGTTTAGCCAAG-3′ (Reverse). Sequencing results were obtained from the University of Calgary Core DNA services. The sequence that was established, including the premature stop codon in the *S1* gene (Table 1), has remained stable for 3 years of culture since the AV virus was first isolated from HTR1 cells.

### Immunoblot, radiolabelling, FACS and electron microscopy

Cell lysates were prepared by sonication in a buffer containing 10 mM Tris (pH 7.4), 2 mM EDTA, 1% NP-40, 50 mM mercaptoethanol, 100 *μ*g ml^−1^ leupeptin and 2 *μ*g ml^−1^ aprotinin. The lysates were then cleared by centrifugation at 16 000 *g* for 15 min, normalised for protein amount, mixed with SDS sample buffer, boiled for 5 min and stored at −70°C. After separation by SDS–PAGE, proteins were transferred to nitrocellulose or PVDF membranes and detected by immunoblot hybridisation. The primary antibodies (Abs) used were: anti-reovirus polyclonal Ab (Strong *et al*, 1998) and anti-*σ*1 N-terminus monoclonal Ab (Duncan and Lee, 1994). Horseradish peroxidase-conjugated anti-mouse Ab or horseradish peroxidase-conjugated anti-rabbit Ab were used as secondary Abs (Pierce Biotechnology, Rockford, IL, USA).

For metabolic labelling, [^35^S]-methionine was added to the culture medium for 12 h. Cells were harvested and lysed in PBS buffer containing 1% Triton X-100, 0.5% sodium deoxycholate and 1 mM EDTA. Lysates were cleared of debris by centrifugation and supernatants were stored at −70°C until use. Polyclonal rabbit anti-reovirus serotype 3 serum was used for immunoprecipitation of [^35^S]-methionine-labelled reovirus proteins from cell lysates as described previously (Lee *et al*, 1981). Immunoprecipitated proteins were subjected to SDS–PAGE, followed by autoradiography.

For flow cytometry analysis, cells were trypsinized and fixed using cytofix/cytoperm solution (PharMingen, San Diego, CA, USA). The fixed and permeabilized cells were incubated with primary reovirus antiserum and secondary FITC-conjugated anti-rabbit IgG (Cedarlane, Burlington, ON, Canada), then analysed by flow cytometry.

### *In vitro* transcription and translation

Trizol was used to extract dsRNA from purified AV reovirus. The resulting RNA underwent cDNA synthesis using the *S1* gene-specific primer (5′-GATGAAATGCCCCAGTGCCGC-3′) and the conditions for SuperScript II reverse transcriptase (Invitrogen). The cDNA was used as template for PCR amplification of AV reovirus S1. Using primers 5′-AGGAATTCGCTATTGGTCGGA-3′ (Forward) and 5′-GCTAGCTAGAGCGGCCGCGATGAAAT-3′ (Reverse), AccuPrime *Pfx* DNA polymerase (Invitrogen), and an annealing temperature of 55C, the S1 open reading frame was amplified with *Eco*R1 and *Not*1 restriction enzyme overhangs. The S1 PCR product was PCR purified (Invitrogen) and double-digested at 37°C for 2 h along with vector, pcDNA3 (Invitrogen), using restriction enzymes *Eco*R1 and *Not*1 (NEB, Pickering, ON, Canada). Digested products were gel purified (Invitrogen) and then ligated (Invitrogen) at room temperature for 1 h. The AV reovirus S1 plasmid was transformed into Top10 *Escherichia coli*-competent cells and screened by ampicillin resistance. Individual bacterial colonies were inoculated into ampicillin LB broth, midi-prepped (Invitrogen) and analysed for incorporation of the S1 insert.

Using the AV reovirus S1 plasmid as template, the AV S1 open reading frame was amplified by *Taq* DNA polymerase (Invitrogen) at an annealing temperature of 55°C using the primers 5′-GGTACCTAATACGACTCACTATAGGGGCTATTGCCACC-3′ (Forward) and 5′-GATGAAATGCCCCAGTGCCGC-3′ (Reverse), which were optimised for T7 transcription. The S1 PCR product was gel purified (Invitrogen) and eluted using 10 mM Tris pH 8.0. *In vitro* transcription was performed using a 50 *μ*l reaction containing 5 *μ*g of AV reovirus S1 template by the RiboMAX large-scale RNA production systems for T7 polymerase (Promega, Nepean, ON, Canada). The transcription reaction was treated with DNase (Promega) for 10 min at 37°C to remove any residual DNA template, RNA purified (Qiagen, Mississauga, ON, Canada), and then analysed by RNase-free agarose gel electrophoresis. The AV reovirus S1 RNA transcript was translated using a 25 *μ*l reaction from the rabbit reticulocyte lysate system (Promega). The samples were analysed by 12% SDS–PAGE and subsequent autoradiography.

### Animal studies

Severe combined immunodeficiency mice (Charles River, Wilmington, MA, USA) received a single subcutaneous implantation of 5 × 10^6^ cells of murine ESCs (R1 or MES1). At 12 days after implantation, palpable teratomas were observed. The R1 teratomas were then injected with reoviruses (WT reo (wild-type reovirus), 1 × 10^7^ PFU/tumour; *n*=5, AV reo (AV reovirus), 1 × 10^7^ PFU/tumour: *n*=5, D reo (Dead, UV-inactivated reovirus): *n*=5) and teratoma growth was followed 21–70 days post-infection. For the MES1 teratoma experiment, 12–15 days after implantation, teratomas were intratumourally injected with reoviruses or PBS (WT Reo (wild-type reovirus, 10^7^ PFU per mouse); *n*=3, AV Reo (AV reovirus, 10^7^ PFU per mouse): *n*=3, PBS: *n*=3) and teratoma growth was followed 31–46 days post-infection.

Each SCID mouse received single subcutaneous implantations of 5 × 10^6^ cells of HT1080 or HCT116 cells suspended in PBS. At 11 days after implantation, 4–6 mice with tumours were injected intratumourally with WT, AV or D (Dead, UV-inactivated) reovirus at 10^7^ PFU/tumour and tumour growth was followed up to 34 days post implantation. WT reovirus was reinjected after 23 days post implantation and AV reovirus was reinjected after 23 and 27 days.

All the mice were treated according to the protocols approved by the University of Calgary Animal Care Committee. Tumour growth was measured externally using calipers and the volume was determined by the equation V=(L × W^2^) × 0.5, where L is the largest dimension and W is the largest dimension perpendicular to L. Tumours and hearts were taken from the mice at 34–35 days post implantation and fixed in 10% buffered formalin solution at room temperature, then paraffin embedded. Teratomas were taken from the mice at 21–70 days post-infection, excised and fixed in 10% buffered formalin solution at room temperature. The specimens were processed by the University of Calgary Histopathology Laboratory Research Service for routine histological analysis (H&E staining).

### Histology and Immunohistochemistry

For reovirus antigen detection (reovirus structural proteins), deparaffinized tumour sections were retrieved in a solution containing 50 mM Tris (pH 7.5), 120 mM NaCl, 0.2% Tween 20 and 0.1% Triton X-100. After blocking the sections with a solution containing 50 mM Tris (pH 7.5), 120 mM NaCl, 0.2% Tween 20, 0.1% Triton X-100 and 2% normal goat serum for 1 h, the sections were immunostained with a solution containing 0.1% reovirus antiserum, 50 mM Tris (pH 7.5), 120 mM NaCl, 0.2% Tween 20, 0.1% Triton X-100 and 2% normal goat serum for 2 h. As a secondary antibody, biotinylated goat anti-mouse antibody (Vector Laboratories, Burlingame, CA, USA) was used at 1 : 100 in a solution containing 50 mM Tris (pH 7.5), 120 mM NaCl, 0.2% Tween 20, 0.1% Triton X-100 and 2% normal goat serum for 2 h at room temperature. Detection was monitored by a diaminobenzidine tetrahydrochloride-based immunohistochemistry protocol according to the manufacturer (Vector Laboratories). Dehydration was carried out in a series of graded ethanol solutions, followed by clarification in xylene. Slides were mounted with Vectamount (Vector Laboratories) and stored at 25°C.

## Results

### Persistent reovirus infection of cultured cells gives rise to reovirus variants

**Figure 1:**
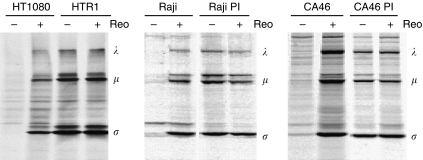
Persistent infection of reovirus in various cancer cells. HT1080, Raji and CA46 cells and their persistently infected derivatives were infected with wild-type reovirus (MOI of 40) and labelled with [^35^S]-methionine at 3 hpi for 12 h. At 24 hpi, cells were lysed, immunoprecipitated with anti-reovirus antibodies, and subjected to SDS–PAGE and autoradiography. Reovirus protein classes are indicated by *λ*, *μ* and *σ*.

Our group recently reported that persistent reovirus infections could be established in oncogenic N-Ras human fibrosarcoma HT1080 cells and p53 mutated lymphoma cells, yielding persistently infected (PI) HTR1 (Kim *et al*, 2007) and Raji PI (Alain *et al*, 2006) cells, respectively. In this study, we have also included a newly established persistent reovirus infection from the Burkitt's lymphoma cell line CA46 (p53 mutated). As shown in Figure 1, infected parental cells exhibited abundant reovirus protein synthesis, whereas PI derivatives showed viral proteins (*λ*, *μ* and *σ* classes) even without the addition of wild-type reovirus. Furthermore, the viral protein expression profile in the PI cells was similar to that of the newly infected parental cells.

### AV reovirus produces a truncated *σ*1 protein

Virus preparations from each of the infected cell lines were analysed for genetic modifications. As alterations of the *S1* and *S4* genes have a role in persistently infected L cells (Ahmed and Fields, 1982; Wetzel *et al*, 1997), gene segments from HTR1, Raji PI and CA46 PI-derived reoviruses were sequenced. As shown in Table 1, multiple mutations of *S1* and *S4* genes were detected. Surprisingly, the S1 gene of the HTR1-derived virus contained a stop codon mutation at amino acid 251 in the *S1* gene product (cell attachment protein *σ*1). *σ*1 is responsible for binding host cell receptors, including primary receptor sialic acid and various secondary receptor proteins (Lee *et al*, 1981; Chappell *et al*, 1997; Barton *et al*, 2001).

**Figure 2:**
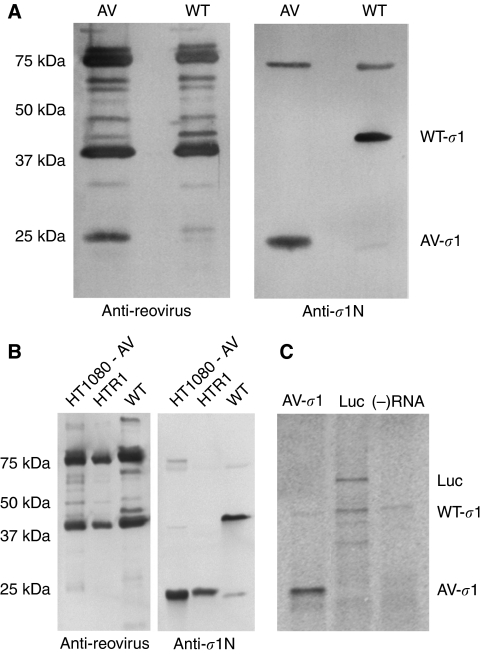
AV reovirus possesses a truncated *σ*1 protein. (**A**) WT and AV reovirus were purified from L929 murine fibroblasts and examined for the presence of *σ*1. Through immunoblotting, membranes were probed with reovirus antiserum (left panel) and polyclonal reovirus *σ*1 N-terminus antiserum (right panel). The N-terminal antibody was raised against *σ*1 amino acids 1–158 (Duncan and Lee, 1994). (**B**) Attenuated reovirus was collected from infected HT1080 and PI HTR1 supernatants by high-speed ultracentrifugation and examined by immunoblotting using reovirus antiserum (left panel) and polyclonal *σ*1 N-terminus antiserum (right panel). Purified WT reovirus was used as a point of reference. (**C**) *In vitro* transcription of the AV reovirus *S1* gene by T7 polymerase produced a *σ*1 RNA transcript that was *in vitro* translated using a rabbit reticulocyte system. Translation of a luciferase transcript and a minus RNA reaction were used as positive and negative controls, respectively. The samples were analysed by 12% SDS–PAGE and subsequent autoradiography.

Immunoblotting of the HTR1-derived reovirus and WT reovirus using a polyclonal anti-reovirus antibody and a polyclonal anti-*σ*1 antibody (raised against the N-terminus) revealed the presence of a 25 kD protein in the reovirus variant; a protein product approximately half the size of its full-length, 455 amino-acid form (Figure 2A). HT1080 cells infected with the HTR1 reovirus variant were also found to express the truncated *σ*1 protein (Figure 2B). Furthermore, *in vitro* transcription and translation of the cloned *S1* gene from the HTR1-derived virus also generated a truncated 25 kD *σ*1 protein (Figure 2C). Collectively, these results confirm that the PI HTR1-derived reovirus variant produces a truncated *σ*1 protein representing the N-terminal half of the full-length *σ*1 protein and from which the C-terminal portion (which is active in cell adhesion; Weiner *et al*, 1977; Wilson *et al*, 1996; Wetzel *et al*, 1997) is absent. The virus variant was accordingly referred to as AV reovirus.

### AV reovirus spares the developmental potential of normal mESCs

**Figure 3:**
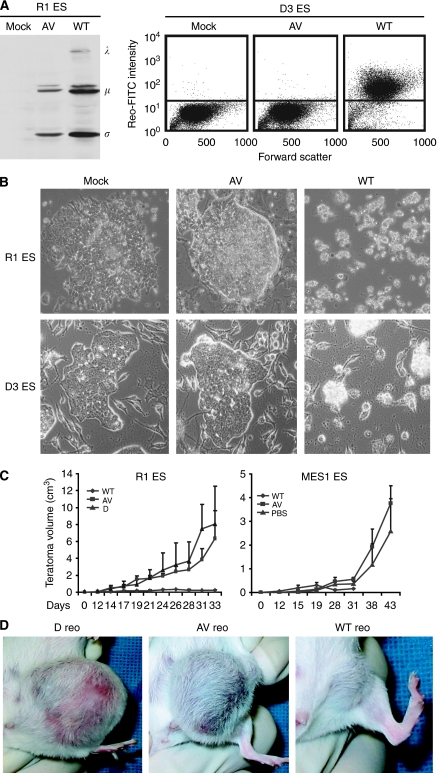
Cytopathogenicity of AV and WT reovirus on embryonic stem cells. (**A**) Pluripotent murine ESCs (R1 and D3) were infected with WT or AV reovirus (MOI of 20). At 48 hpi, cell lysates were prepared and viral proteins (*λ*, *μ* and *σ*) were detected by immunoblotting using reovirus antiserum (left panel). Alternatively, cells were fixed/permeablized for FACS analysis at 48 h post-infection and reovirus antiserum and secondary FITC antiserum were used to detect the presence of reovirus proteins (right panel). (**B**) At 48 hpi, viral cytopathic effects were also photographed. (**C**) Severe combined immunodeficiency mice received a single implantation of murine R1 (left panel) or MES1 (right panel) ESCs. At 12 days following implantation, teratomas were intratumourally injected with WT or AV reoviruses (10^7^ PFU per mouse; *n*=3–5) or D Reo (Dead, UV-inactivated reovirus) or PBS and teratoma growth was monitored for 21–70 days post-infection. Mice treated with WT reovirus were killed at 18–19 days post-infection because of the formation of viral-induced myocarditis. (**D**) Photographs of representative R1 teratomas were taken 21 days after implantation (9 days post-infection).

In addition to causing various pathogenic manifestations in immunodeficient hosts, WT reovirus is also known to rapidly target normal ESCs and adversely affect the formation of rat and mouse blastocysts; thus inhibiting embryonic development (Heggie and Gaddis, 1979; Priscott, 1983). To determine if the attenuated virus was capable of inducing cytopathogenicity in ESCs, two murine ESC lines (R1 and D3) were infected with either AV or WT reovirus. In comparison with the WT virus, immunoblot and flow cytometry analyses revealed minimal infectivity of ESCs by AV reovirus and reduced viral protein synthesis by 48 h post-infection (Figure 3A). In addition, AV reovirus infected R1 and D3 ESCs showed no detectable signs of viral cytopathogenicity, whereas WT reovirus induced extensive cytopathic effects and the destruction of embryoid bodies (Figure 3B). To compare AV and WT reovirus pathogenesis *in vivo*, a murine teratoma model was adopted, in which pluripotent ESCs were able to develop into mature benign teratomas with a high level of differentiation upon xenograft implantation *in vivo* (Sasaki *et al*, 2005). Thus, SCID mice were xenografted with murine R1 or MES1 ESCs and teratomas were allowed to develop into palpable tumours. At 12 days post implantation, AV or WT reoviruses were delivered intratumourally and benign teratoma growth was monitored (Figure 3C). Photographs of representative R1 teratomas were taken 9 days after reovirus administration (21 days after implantation) (Figure 3D). Consistent with the *in vitro* observations, AV reovirus did not suppress benign teratoma growth or differentiation, whereas WT reovirus prevented further teratoma growth and induced neuroglia necroses (histological data not shown), supporting previous findings that WT reovirus targets the nervous system in suckling mice (Flamand *et al*, 1991; Oberhaus *et al*, 1997). Therefore, unlike WT reovirus, AV reovirus exhibits a reduced cytopathogenicity towards various non-cancerous and differentiated cell types.

### AV reovirus retains oncolytic activity *in vitro*

**Figure 4:**
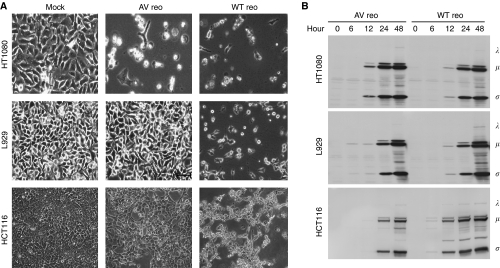
AV reovirus retains its oncolytic activity *in vitro*. (**A**) HT1080, L929 and HCT116 cells were either mock infected or infected with WT or AV reovirus at an MOI of 40. At 48 h post-infection, viral cytopathic effects were photographed. (**B**) HT1080, L929 and HCT116 cells grown to 70% confluency were infected with WT or AV reovirus at a MOI of 20. Cell lysates were collected at the indicated timepoints and using equal loading, were analysed by immunoblotting with polyclonal reovirus antiserum.

As the *S1* gene of reovirus has a critical role in binding host cells and in virus-induced apoptosis (Lee *et al*, 1981; Duncan *et al*, 1991; Tyler *et al*, 1995; Connolly *et al*, 2001), we speculated that the oncolytic activity of AV reovirus would be somewhat reduced. To determine if AV reovirus mimicked the wild-type form by preferentially targeting Ras-transformed cells, N-Ras-transformed HT1080 fibrosarcoma cells and K-Ras-transformed HCT116 colon carcinoma cells were challenged with the virus. Attenuated reovirus retained its ability to kill parental HT1080 cells efficiently *in vitro*, however its pathogenesis on L929 and HCT116 cells was somewhat reduced (Figure 4B). Cytopathic effects on L929 cells by AV reovirus were observed by 72 hpi (data not shown). Attenuated reovirus protein synthesis remained comparable or slightly decreased compared with the WT virus, suggesting that the cytolytic activity of AV reovirus had been partially attenuated (Figure 4B). Furthermore, our group recently showed that cancer cells dysfunctional in p53, ATM or Rb, are susceptible to both WT and AV reovirus (Kim *et al*, 2010). Thus, although the cytocidal potency of AV reovirus *in vitro* is somewhat reduced, it retains its ability to infect and replicate in a variety of tumour cell types.

### AV reovirus displays reduced host toxicity while retaining its oncolytic activity *in vivo*

Even though WT reovirus infection is often asymptomatic or causes only mild disease in immunocompetent hosts, it can induce various pathologic manifestations, such as myocarditis, vasculitis, black tail syndrome and neuronal damage in newborn and immunodeficient animals (Sabin, 1959; Weiner *et al*, 1977; Baty and Sherry, 1993; Terheggen *et al*, 2003; DeBiasi *et al*, 2004; Loken *et al*, 2004). Genetic reassortment studies of reovirus genes have implicated S1 in having a significant role in determining reovirus pathogenesis (Weiner *et al*, 1977, 1980; Haller *et al*, 1995). As the replication competent AV reovirus is mutated in the *S1* gene, produces a truncated *σ*1 protein, and exhibits reduced cytolytic potential *in vitro*, its capacity for oncolysis and pathogenesis *in vivo* was also assessed.

**Figure 5:**
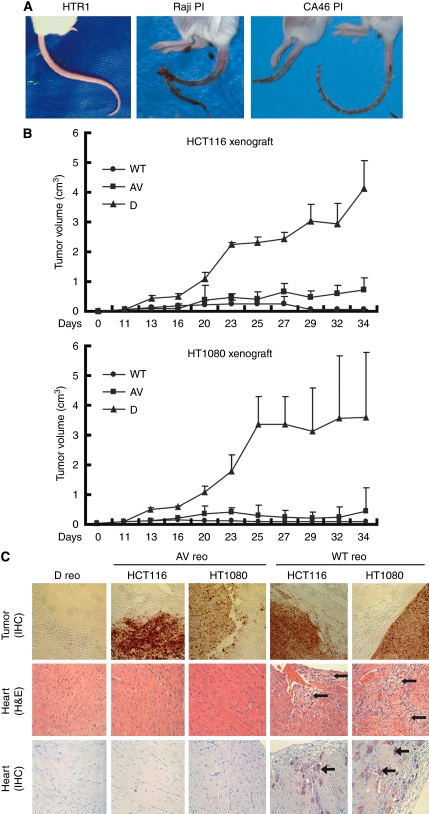
AV reovirus remains oncolytic *in vivo* and shows reduced toxicity. (**A**) HTR1, Raji PI and CA46 PI cells were injected into the left flanks of SCID mice, which were photographed after 3–4 weeks and as morbidity developed. All Raji PI- and CA46 PI- treated mice developed black tails at 22–25 days post-injection (center and right panels) and were killed shortly thereafter; whereas HTR1-treated mice displayed no signs of distress after 4 weeks (left panel) and only began to develop black tails at 3–7 months post-injection. (**B**) Severe combined immunodeficiency mice received single implantations of 5 × 10^6^ HCT116 colon carcinoma or HT1080 fibrosarcoma cells. At 11 days after implantation, palpable tumours were injected with WT, AV or UV-inactivated (Dead; D) reoviruses at 10^7^ PFU/tumour and tumour growth was followed up to 34 days post-implantation. WT reovirus was reinjected 23 days post-implantation and AV reovirus was reinjected 23 and 27 days post-implantation. (**C**) Histological comparison of HCT116 and HT1080 tumours and heart tissue from SCID mice treated with reoviruses (WT, AV and D reovirus as above). Paraffin sections of reovirus-treated tumours (24 days post-infection) were analysed by indirect immunohistochemical staining using reovirus antiserum; brown staining represents reoviral antigen positive regions (upper panels). Paraffin sections of heart tissue from reovirus-injected mice (23 days post-infection) were analysed by H&E (middle panels) and indirect immunohistochemical staining (lower panels) using reovirus antiserum. Extensive necrotic lesions (arrows) and massive lymphocyte infiltration were observed in heart tissue from WT reovirus-infected mice, but not AV virus-treated mice (middle panel). Wild-type reovirus infected mice also showed reoviral antigen-positive regions (arrows; lower panel); sections were counter-stained with methyl green.

We first compared the AV reovirus producing cell line, HTR1, with two other reovirus persistently infected cells lines (Raji PI and CA46 PI) for their respective pathogenicities in reovirus-susceptible SCID mice (George *et al*, 1990; Loken *et al*, 2004). Previously, we showed that persistently infected cells lose tumourigenicity *in vivo* (Alain *et al*, 2006; Kim *et al*, 2007), and as expected, none of the three persistently infected cell lines were able to produce tumours in SCID mice. Nevertheless, mice injected with Raji PI or CA46 PI cells rapidly developed black tail syndrome (Figure 5A) and displayed severe morbidity by 3–4 weeks post-inoculation. In contrast, HTR1 inoculated mice showed no visible morbidity after 1 month and only began to develop black tail syndrome after 3–7 months.

To assess the oncolytic potential of AV reovirus *in vivo*, human Ras-oncogenic HCT116 colon carcinoma cells or HT1080 fibrosarcoma cells were implanted in SCID mice and subsequently treated with reovirus. During the first 23 days of virus treatment, it was found that both WT and AV reovirus effectively suppressed tumour growth, whereas inactivated virus (D reo) showed no suppression of tumour growth (Figure 5B). At this point, it was clear that tumour suppression by AV or WT reovirus was comparable, however, it did not address the issue of host cytotoxicity and as a result, longer-term studies were required. As current clinical trials utilise multiple reovirus administrations, AV reovirus was reinjected two more times at day 23 and 27 into the established SCID mice tumours. Wild-type reovirus was also reinjected at day 23, but not at day 27 because of increased host cytotoxicity. This is consistent with previous studies that have already shown that a single injection of the WT virus is enough to cause black tail syndrome in SCID mice (Loken *et al*, 2004). Ultimately, mice treated with WT reovirus were all killed at 25–40 days post-viral inoculation because of severe morbidity (weight loss and black tail development), whereas mice treated with AV reovirus only developed these pathologies after several months post-treatment. Immunohistochemical analysis revealed that both WT and AV reovirus replicated well at tumour sites (Figure 5C).

As WT reovirus is known to cause myocarditis (Baty and Sherry, 1993; Terheggen *et al*, 2003), heart tissue was examined by hematoxylin and eosin staining, and analysed by immunohistochemistry. Mice treated with WT reovirus showed distinct signs of viral myocarditis, as severe cardiac muscle damage and massive lymphocytic infiltration were observed (Figure 5C). On the other hand, mice treated with AV reovirus showed no symptoms of heart damage by 25 days post-viral treatment. Immunohistochemical analysis of heart tissue revealed an abundance of viral proteins in mice treated with WT reovirus, whereas no viral proteins were detected in the heart tissues of mice treated with AV reovirus (Figure 5C). Taken together, WT reovirus not only infected tumour sites and prevented tumour growth, but also systemically infected host heart tissues. In contrast, AV reovirus retained efficient oncolytic activity and caused less damage to healthy tissue in the SCID mouse model.

## Discussion

As a naturally oncolytic virus, reovirus preferentially infects and kills cancer cells with active Ras signalling or defective tumour suppressors, making the virus a promising anti-cancer agent (Coffey *et al*, 1998; Kim *et al*, 2010). In light of this finding, reovirus has been tested in a wide range of cancers both *in vitro* and *in vivo*, and is currently undergoing phase I, II and III clinical trials (Strong *et al*, 1998; Duursma and Agami, 2003; Norman *et al*, 2004). Although only mild toxicity has been reported in patients (Thirukkumaran and Morris, 2009), animal models have indicated that reovirus pathogenesis is not entirely restricted to cancer cells, as the virus is capable of inducing haemorrhage, fibrosis, hepatitis, pancreatitis, necrotising encephalitis and myocarditis in mice (Sabin, 1959; Baty and Sherry, 1993; Richardson *et al*, 1994; Mann *et al*, 2002; Loken *et al*, 2004). Furthermore, as these viruses are delivered to tumour-bearing hosts by intravenous injection, normal tissues, including stem cells and their developmental potential, may be subject to viral challenge (Heggie and Gaddis, 1979; Priscott, 1983). It may therefore be useful to identify and generate a modified reovirus that exhibits enhanced safety while maintaining its oncolytic potential, thereby providing a potentially higher dose or treatment number.

During our study of reovirus resistance in Ras-transformed human fibrosarcoma HT1080 cells (Kim *et al*, 2007), we serendipitously isolated an S1 attenuated reovirus variant from persistently infected HTR1 cells (Figure 1). As is the case with reovirus mutants derived from persistent infections, the AV reovirus arising from the HTR1 culture displayed significant genetic alterations. Genomic sequence analysis revealed several mutations in the *S1* gene segment, with the most significant resulting in a nonsense mutation at nucleotide 763 and a truncated *σ*1 protein (Table 1 and Figure 2). It is noteworthy that the sequence that was established for the AV reovirus, including the premature stop codon in the *S1* gene, has remained stable for over 3 years of culture, suggesting a highly stable coadaptation between virus and host cell in the HTR1 culture (Kim *et al*, 2007).

Genetic reassortment studies have shown that the *S1* gene segment of reovirus T3D is the major determinant in reovirus-induced pathogenesis (Weiner *et al*, 1977, 1980; Dichter and Weiner, 1984; Haller *et al*, 1995), with the *σ*1 protein having a critical role in reovirus-mediated apoptosis (Tyler *et al*, 1995; Connolly *et al*, 2001). Despite the S1 modification, AV reovirus retained robust viral replication in various cancer cell types, including those with an activated Ras signalling pathway (Figure 4). Although the AV virus displayed a significantly reduced, but not abolished, apoptotic potential *in vitro* (Figure 4), it was able to reduce tumour size *in vivo* to levels comparable with that of the WT virus (Figure 5). In terms of its pathogenesis in normal cells, AV reovirus consistently caused less damage to normal cells than its WT counterpart. The virus only minimally infected ESCs *in vitro* and did not adversely affect ECS development *in vivo* (Figure 3). Furthermore, even with multiple injections in SCID mice, AV reovirus did not induce black tail syndrome or myocarditis (Figure 5). Taken as a whole, AV reovirus pathogenesis is limited to cancer cells and this oncolytic specificity is even greater than the WT virus when taking into account the reduced damage by AV virus to normal cells.

Unlike the AV reovirus from HTR1, reoviruses from Raji PI and CA46 PI, both possess full-length *σ*1 and caused severe viral pathogenesis comparable with that of WT reovirus (Loken *et al*, 2004). This was consistent with the notion that the reduced viral pathogenesis seen with AV reovirus was strongly associated with the *S1* gene attenuation acquired during persistent reovirus infection of the HT1080 fibrosarcoma cells, but not necessarily arising during the persistent infection of other cell types. As AV reovirus also contained several mutations in the *S4* gene (S4 mutations are also present in CA46 PI- and Raji PI-derived viruses), which encodes one of the major outer capsid proteins, we cannot rule out the possibility that these mutations (and those that may be present in other gene segments) also have a role in virus attenuation. However, in view of the well-documented role of the *S1* gene in reovirus pathogenesis, we favour the idea that the nonsense mutation in S1 likely has a major, if not the sole, role in AV virus attenuation.

With its significantly reduced viral pathogenic potential and retained ability to display strong oncolytic activity *in vivo*, AV reovirus may deserve consideration as an alternative therapeutic agent for various types of tumours in immunosuppressed patients. These findings may also serve as a starting point for optimising reovirus’ potential as a cancer therapeutic through the use of the newly developed reverse genetics system for dsRNA viruses (Kobayashi *et al*, 2007).

## Figures and Tables

**Table 1:** *S1* and *S4* gene mutations of persistently infecting reoviruses

		**Mutation position**		
**Persistent reovirus**	**Reovirus gene**	**Nucleotide**	**Amino acid**	**GenBank accession no.**
HTR1	*S1*	359, T–C	116, L–P	AY860061
		392, T–C	127, V–A	
		763, C–T	251, Q–Stop	
		912, A–G	300, I–M	
	*S4*	562, C–T	177, S–F	DQ915165
		784, A–T	251, H–L	
CA46 PI	*S1*	89, A–C	26, K–T	EF133509
			7, K–Q	
		1351, A–T	447, T–S	
	*S4*	720, C–T	230, H–Y	EF133510
		730, C–T	233, S–L	
		1089, A–C	353, N–Q	
		1091, T–G	353, N–Q	
		1092, T–C	354, Y–H	
Raji PI	*S1*	—	—	EF133511
	*S4*	784, A–T	251, H–L	EF133512

Mutations in *S1* and *S4* gene nucleotide sequences of persistently infecting reoviruses from HTR1, Raji PI and CA46 PI cells, and corresponding mutations in the deduced amino acid sequences of their gene products (S1: *σ*1/*σ*1 s, S4: *σ*3) compared with previously published sequences of WT reovirus (GenBank accession no: S1 (X01161), S4 (K02739)). As reovirus occasionally undergoes spontaneous genome variations, nucleotide changes that were found in all the persistently infecting reoviruses and in our lab, WT reovirus were not included in this table (S1: 366, C–G, 367, G–C, S4: 624, G–A, 719, G–T).
